# An image-based modeling framework for predicting spatiotemporal brain cancer biology within individual patients

**DOI:** 10.3389/fonc.2023.1185738

**Published:** 2023-10-02

**Authors:** Kamila M. Bond, Lee Curtin, Sara Ranjbar, Ariana E. Afshari, Leland S. Hu, Joshua B. Rubin, Kristin R. Swanson

**Affiliations:** ^1^ Mathematical Neuro-Oncology Lab, Department of Neurological Surgery, Mayo Clinic, Phoenix, AZ, United States; ^2^ Hospital of University of Pennsylvania, Department of Neurosurgery, Philadelphia, PA, United States; ^3^ Department of Radiology, Mayo Clinic, Phoenix, AZ, United States; ^4^ Departments of Neuroscience and Pediatrics, Washington University School of Medicine, St. Louis, MO, United States

**Keywords:** glioblastoma, radiomics, machine learning, MRI, imaging, CNS tumor, personalized medicine, glioma

## Abstract

Imaging is central to the clinical surveillance of brain tumors yet it provides limited insight into a tumor’s underlying biology. Machine learning and other mathematical modeling approaches can leverage paired magnetic resonance images and image-localized tissue samples to predict almost any characteristic of a tumor. Image-based modeling takes advantage of the spatial resolution of routine clinical scans and can be applied to measure biological differences within a tumor, changes over time, as well as the variance between patients. This approach is non-invasive and circumvents the intrinsic challenges of inter- and intratumoral heterogeneity that have historically hindered the complete assessment of tumor biology and treatment responsiveness. It can also reveal tumor characteristics that may guide both surgical and medical decision-making in real-time. Here we describe a general framework for the acquisition of image-localized biopsies and the construction of spatiotemporal radiomics models, as well as case examples of how this approach may be used to address clinically relevant questions.

## Introduction

While great strides have been made in elucidating the biology of central nervous system tumors, our understanding of these diseases is far from complete. Brain tumors, including but not limited to glioblastoma (GBM), are known to have profound variability between patients (1–4). Intratumoral heterogeneity is rampant as well, and single-cell studies have identified genotypically and phenotypically distinguishable cellular subtypes even within the same sample (5–8). Regional nuances in a tumor’s microenvironment add another layer of complexity, as two biopsies with the same genotype may have completely different phenotypes and therapeutic sensitivities (9). The ability to train machine learning models to predict these biological characteristics simply from MR images (i.e. radiomics) would allow us to assess a tumor in its totality and track changes over time. This would be immensely valuable in the context of CNS malignancies, where tissue sampling is limited due to the tumor’s eloquent location and biopsies are unlikely to be representative of the entire landscape.

Since imaging is a routine component of tumor surveillance and treatment monitoring, the scalability and value of radiomics models are enormous. Thus far, the majority of image-based models for the evaluation of brain tumors have focused on classifying the genomics across the whole tumor or large regions within the tumor. For example, several studies have used standard MRI sequences (10–12), dynamic susceptibility contrast MRI (13, 14), and diffusion MRIs (15–17) to discriminate between IDH wildtype and IDH mutant gliomas. Similar approaches have been used to predict MGMT promoter methylation status (18–23), transcriptomic subtypes (24, 25), and classify patients as short-, mid-, and long-term survivors (26). By definition, these whole-tumor approaches overlook the genomic, transcriptomic, and microenvironmental heterogeneity that is known to exist within and between tumors (3–9).

The ability to predict regional changes on a voxel-by-voxel level has immense potential value, as it would allow for the complete characterization of intratumoral heterogeneity in the absence of tissue sampling. However, spatial models are uniquely challenging to build. Without knowing exactly where a sample was harvested from, biological and radiographic data cannot be aligned in a meaningful way. To overcome this limitation, intraoperative surgical navigation can be used to localize a biopsy’s coordinates on MRI. In this way, a tissue sample’s imaging features and characteristics from secondary biological tests (e.g. genomics, RNA sequencing, etc.) can be combined to train machine learning models that establish connections between tumor biology and MRI. This approach can be applied to large cohorts to measure inter-patient variability, as well as serial imaging from the same patient to assess intratumoral dynamics over time. This voxelwise approach has been utilized (20, 27–29), though to a lesser extent, due to the more tedious nature of the data collection. Here we provide a framework for collecting image-localized biopsies and leveraging machine learning to build spatial radiomics models. We also provide case examples that demonstrate the clinical potential of these spatiotemporal models.

## Framework to generate image-based machine learning models (Methods)

**Figure 1:**
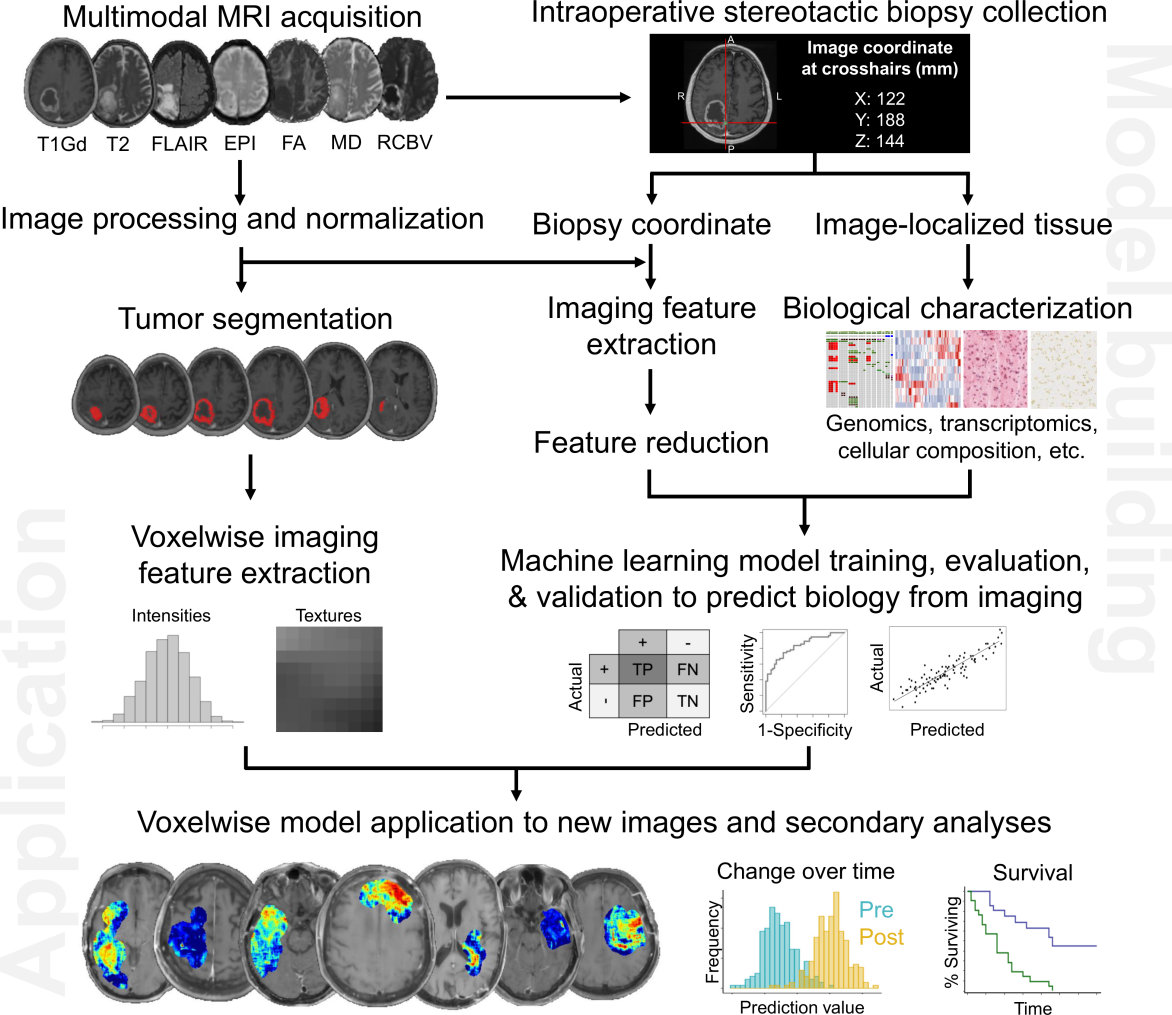
Pipeline to generate image-based machine learning models that predict voxelwise intratumoral biology from MRI. As summarized here, these maps can reveal aspects of tumor biology that can be used to guide diagnosis (e.g. specific genomic characteristics), treatment planning (e.g. extent of surgical resection to minimize residual tumor cell burden), and even treatment assessment by tracking key biological attributes over time.

Any imaging modality (e.g. CT, MRI, PET) can be used to extract imaging features for training machine learning algorithms. Since MRI is the clinical gold standard for patients with CNS tumors, we will discuss a pipeline for the acquisition and standardization of MRIs, generation of machine learning models that predict biological characteristics from MRI, and ultimately the prediction of biology from new, unseen images for secondary analysis (**Figure 1**).

### Multiparametric MRI and image-localized biopsies

Before surgery, each patient undergoes conventional multimodal MR imaging (T1-weighted with Gadolinium-based contrast agent, T2-weighted, and fluid-attenuated inversion recovery sequences). Spoiled gradient recalled-echo (SPGR) images are obtained for use during surgical navigation. Advanced sequences are obtained when possible (e.g. perfusion, diffusion tensor imaging, echo-planar imaging).

Intraoperative navigation loads SPGR and T2/FLAIR images to stereotactically guide tumor biopsy and/or resection. While several external landmarks are used to check navigation accuracy preoperatively, registering a patient’s preoperative images to their intraoperative anatomy introduces a risk of measurable error. Immediately preceding tissue collection, surgeons attempt to validate the accuracy of the navigation system with nearby anatomic landmarks (i.e., skull, vasculature, ventricles, etc.). If the navigation and anatomy are aligned with minimal error (<1mm), a biopsy is collected and the coordinates of the biopsy site are recorded with accompanying screenshots of the navigation monitor. Each patient has multiple samples collected with image localization, all of which are either flash-frozen or embedded in formalin for biological testing. The coordinates and screenshots obtained at the time of biopsy harvesting are later co-registered with the full preoperative MR imaging data for further analysis using previously-described methods (30). The accuracy of this collection process is limited by the subjective threshold set by the surgeon and intraoperative research team. At our institution, the perceived error between imaging location and anatomical location must be negligible (<1mm) to qualify for biopsy collection and downstream analyses. If one was studying a less heterogeneous pathology where spatial resolution was of a concern, error thresholding could theoretically be more lenient.

### Image registration and normalization

Registration is the process of geometrically aligning two or more images (or an image with a standardized atlas) such that any given coordinate represents the same location in every image. Many linear and non-linear registration tools exist to perform this task (31). At a minimum, different MRI sequences obtained at a single time point need to be coregistered for accurate feature extraction. While not mandatory, registering longitudinal visit-to-visit series data can be helpful when attempting to visualize changes over the course of treatment.

Conventional MR images have arbitrary units that are not stable across patients, protocols, and scanners. Therefore, image intensities must be normalized to improve the accuracy, test-retest robustness, and generalizability of the radiomics models. There are several mechanisms to standardize image intensities including histogram-based and statistical normalization methods (32, 33). N4 normalization, available in the SimpleITK package, is a popular example of such a bias field correction technique, that makes use of B-spline approximation of the bias field and assumes independent Gaussian noise (34). Z score normalization, White Stripe normalization and Nyul normalization are some other examples (33, 35). Although some recent work has proposed caution in the use of such normalization methods as they can affect the reproducibility of radiomics features (36).

### Segmentation and feature extraction

Segmentation refers to the process of delineating regions of interest (ROI, i.e. biopsy location, contrast-enhancing tumor, or T2/FLAIR-hyperintense penumbra). Biopsy segmentations can be a 2-dimensional square (one axial slice) or a 3-dimensional sphere centered at the imaging coordinate. We formally train individuals to manually segment tumors using in-house software. Segmentations are overseen and approved by a team leader to ensure accuracy and consistency. This manual approach can be tedious and time consuming, but human expertise is better able to discern between complex anatomy (i.e., irregular borders, cysts, skip lesions, ventricle adjacency, etc.) than automated, pattern-based pipelines. Semi-automatic and automatic segmentation protocols are in development and are in high demand. Still, they are still benchmarked against the gold-standard of expert manual segmentation. Comparable manual or semi-automated segmentation can be achieved with most modern personal machines using open-source packages (37).

There are several mechanisms to extract imaging features from MRI sequences. We use PyRadiomics (38), as it can be implemented from the command line to extract the average features across a region (e.g. an entire biopsy ROI) or voxel-by-voxel within a tumor’s segmentation. First- and second-order intensity-based, shape, and texture features can be calculated. All of these values can be extracted before or after applying imaging filters (e.g. Gaussian, Gabor, wavelet). While the number of options for feature extraction is vast, adherence to the Image Biomarker Standardization Initiative guidelines is paramount for the sake of quality control and reproducibility (39).

Choosing the image sequences to include in radiomic analysis is a balance between generalizability and oversimplification. For example, most patients do not routinely obtain complex vascular imaging but do have standard post-contrast T1 and T2/FLAIR images. Thus, training models on features extracted from ten uncommon sequences is extremely unlikely to be adopted in clinical practice nor be able to be validated against historical data sets. On the other end of the spectrum, these models are being asked to find relationships between complex biological features and numeric voxel intensities. In our experience, at least two imaging sequences are needed for models to establish a relationship between these two domains. We tend to start with T1Gd and T2/FLAIR images and make adjustments based on preliminary model performance with these widely available sequences.

### Machine learning

In the context of CNS tumors where biopsies are typically limited due to the eloquence of the brain and spine, the number of imaging features will likely greatly exceed the number of samples. This increases the risk of overfitting, the phenomenon by which a model latches onto the details of the training data at the expense of missing the overall, generalizable pattern. As such, it is imperative to gather as many patients and samples as possible. While there is no universal cutoff for the minimum number needed to train and test either a classification or regression machine learning model, more is better and fewer than 100 samples would raise concerns for generalizability. An imaging feature reduction step must be performed to reduce the likelihood of overfitting, and this step could include: 1) removal of features with zero or near-zero variance, 2) removal of features that are redundant or highly correlated with one another, 3) selection of features that are highly correlated with the target variable, and 4) calculation of variable importance scores.

After feature selection, a machine learning model can be trained to answer the clinical question of interest. Classification models (e.g. discriminant analysis) are trained for the prediction of discrete variables. To predict continuous variables, regression models (e.g. linear regression) should be employed. Some models (e.g. random forest, k-nearest neighbors, support vector machine) can be used in both contexts. Data should be split randomly into training and testing cohorts to estimate the performance of the machine learning models on data not used to train the model. Training data (usually 70% of samples) is used to develop the model, while testing data (the remaining 30%) is used for model validation. The target variable should be represented equally in both the testing and training cohorts.

Machine learning models are assessed by comparing their predictions to ground-truth (“actual”) values. Classification models are assessed using a confusion matrix and receiver operating characteristic curve. Regression models typically report actual versus predicted correlation coefficients, p-values, and root-mean-square error values. A model that performs exceptionally well on training data but poorly on test data suggests that the model may be overfitted and a reassessment of the feature selection may be necessary. Small datasets run the risk of over- or under-estimating model performance based on the way training and test data are split. Under these circumstances, k-fold cross-validation may improve the estimates of model performance by maintaining performance evaluation on unseen small subsets of data and averaging the results into a single, more generalizable performance estimate.

### Pitfalls and challenges

Additional challenges may arise if there is unexpected uncertainty within the image-localization of the samples. While the standard protocol is to locally validate a sample’s location using fixed anatomical reference points, these points may remain navigationally accurate while the brain tissue itself shifts during surgery. This “brain shift” is more common when resecting large lesions that were imposing significant mass effect, so extra care should be taken under these circumstances. In such datasets, one might expand the region within which features are extracted (e.g., increase from a 5x5 region to a 7x7 region to allow for more uncertainty).

Although we strive to include as much data as possible within model training for the sake of generalizability to the clinic, we may occasionally remove patients from analyses if they do not have the necessary imaging required to generate important model features. Similarly, samples may be removed from analyses if there is a high level of uncertainty or technical error (e.g., coordinates not collected, breakdown in communication in the operating room) during their collection (40).

For model generation, collecting the initial cohort for training and validation is by far the rate determining step. To accrue biopsies requires enthusiastic buy-in from partner neurosurgeons as well as significant time, labor, and financial resources for analysis and associated abstraction of longitudinal clinical data. Depending on the model architecture, computational resources may also need to be acquired. Model training can also take some time, especially if using computationally expensive algorithms with parameters that need to be tuned. Adding a further layer of complexity, once a satisfactory model has been created, it is difficult to validate the models using independent datasets because of the inter-institutional heterogeneity in MRI machines, image acquisition protocols, and limitations to data sharing. This lack of reproducibility between institutions is a point of frustration, though many groups are working on approaches for harmonizing images.

## Potential applications and anticipated results

Image-based models can be built to predict almost any biological variable of interest. Of the utmost importance to clinical practice is answering the question, “is this normal or pathologic?” Thus, many clinical models are interested in predicting categorical variables that are binary (e.g. pseudoprogression vs. true tumor progression), nominal (e.g. EGFR deleted, wild type, or amplified), or ordinal (e.g., grade I-IV). Of more interest to basic science and translational research is the ability to predict continuous variables. For example, the percent of malignant cells in a tumor sample, copy number variation, transcript abundance, and percent cellular composition. While the potential biological applications are essentially limitless, we provide a few key examples from the literature in **Table 1**. Further examples about future directions that highlight the utility of spatially informed radiomics are provided below.

**Table 1:** Key examples of clinically-relevant radiomics applications in the literature.

Outcome	Variable Type	Example references
High grade glioma vs. low grade glioma	Binary	Zacharaki et al. (2009) (41), Skogen et al. (2016) (42), Vidyadharan et al. (2022) (43)
Radiation necrosis vs. tumor progression	Binary	Hu et al. (2011) (44), Tiwari et al. (2016) (45), Ismail et al. (2018) (46)
Glioma grade (I-IV)	Ordinal	Xie et al. (2018) (47), Qi et al. (2018) (48)
Patient survival time	Ordinal	Baid et al. (2020) (26), Suter et al. (2020) (49), Chato et al. (2021) (50), Karami et al. (2021) (51)
EGFR mutation status	Nominal	Zinn et al. (2017) (52), Pease et al. (2022) (53)
IDH mutation status	Nominal	Hsieh et al. (2017) (54), Jakola et al. (2018) (55), Lee et al. (2019) (56), Han et al. (2019) (11)

### Diagnosis: genomic alterations

Numerous genetic alterations and their associated signaling pathways have been implicated in tumorigenesis, growth, and invasion (57). While these mutations and copy number variations are challenging to discern histologically, they correlate with tumor subtypes and often reflect the degree of aggression. Several therapies with genetic targets have garnered attention, but their potential for clinical utility hinges upon identifying patients whose tumors harbor these alterations. Surgical biopsies may only be capturing an unrepresentative minority of the entire tumor landscape. Image-based models overcome this limitation by predicting the genetic landscape of the entire tumor. Classification models can be built to predict clonal subpopulations and track their dynamics throughout treatment.

**Figure 2:**
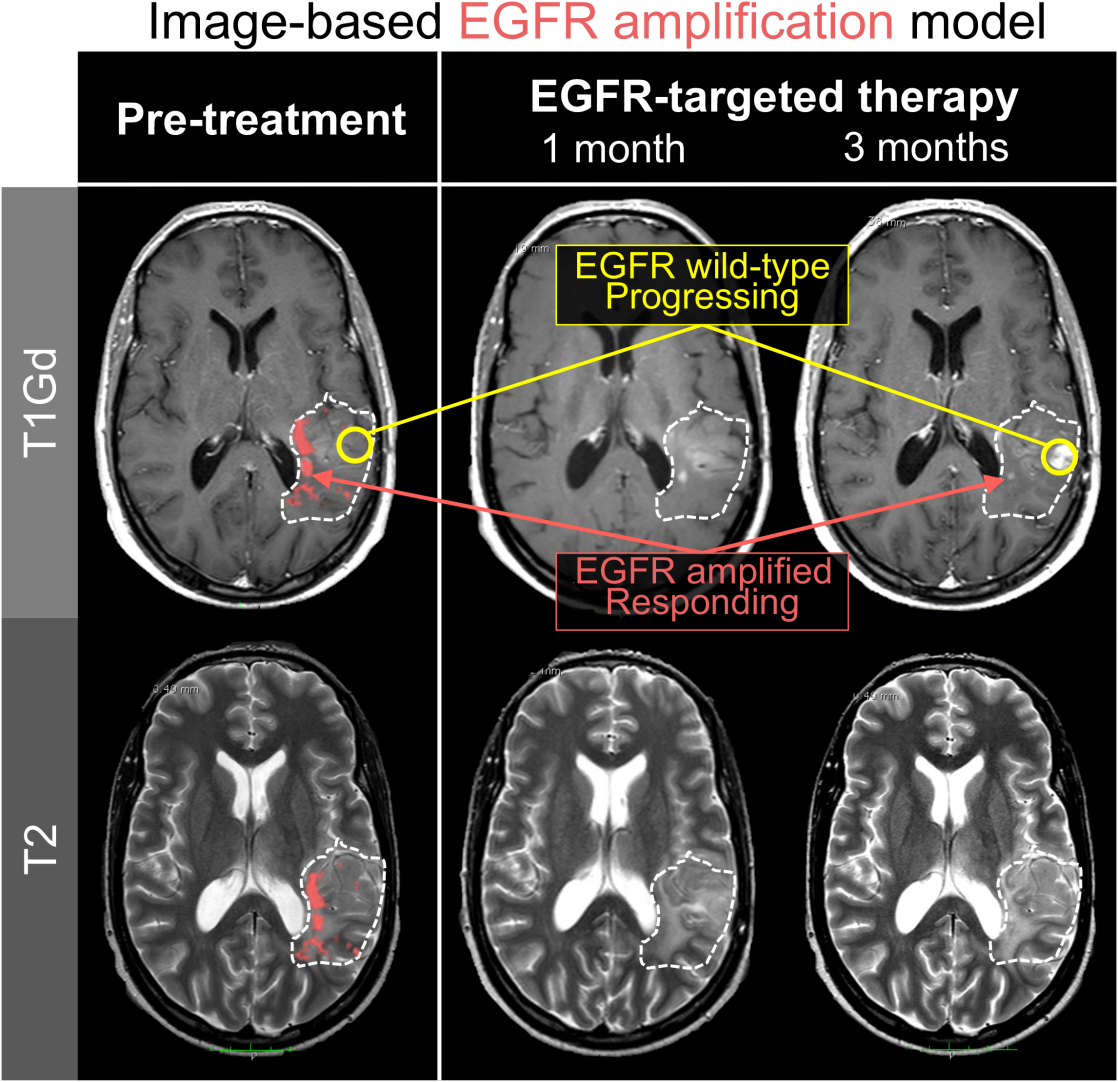
Spatially-resolved radiomics models can predict tumor regions with genomic alterations. In this illustration, an image-based machine learning model predicted EGFR amplification on serial imaging of a patient receiving erlotinib, an EGFR-targeting therapy. Notably, the regions which lose contrast enhancement over the course of therapy are the same regions that were predicted to be EGFR amplified (red). Regions of tumor progression were in locations that the model predicted to be EGFR wild-type (yellow). These results are consistent with the reality that intra-tumoral heterogeneity limits the efficacy of targeted therapies to only subregions within a tumor.

As an example, one could build a model that predicts the presence of CDK4 copy number amplification from imaging. The overexpression of CDK4 induces an oncogenic transition of neural progenitor cells into drivers of tumor growth and progression. This phenotype is known to co-exist amongst other cellular subtypes within a tumor (6). Since CDK4 inhibition is only effective against CDK4-overexpressing tumors, image-based models present the opportunity to identify patients who are uniquely susceptible to these targeted therapies (58, 59). Spatially-resolved models of some genomic aberrations in gliomas (e.g. copy number variations in EGFR, PDGFR, etc.) have already been developed, with accuracies ranging from 37.5% to 87.% depending on the gene of interest (**Figure 2**) (28, 60, 61).

### Surgical planning: tumor cell burden and distribution

By the time a patient receives the formal diagnosis of GBM, tumor cells can already be found throughout the entirety of their brain (62). However, the overall distribution of malignant cells can range from nodular to diffuse (63–65). Together, a tumor’s cellular distribution and the extent of surgical tumor resection have implications for prognosis (64–69). Machine learning models can be trained to predict histology-based estimates of tumor cell density from imaging (27, 70). In some cases, it may be beneficial to supplement machine learning with mechanistic models of tumor biology (71, 72). This can be particularly valuable when biological processes are well defined and machine learning models may not be trained on enough samples to fully detect the known relationship. In a study that compares a machine learning model, a mechanistic model, and a hybrid model, the hybrid outperformed the other two models (p<0.001) and had mean absolute predicted error value less than half of either model alone. Taken together, these approaches provide a means to convert routine clinical imaging into maps of tumor cell density and invasion. Such tools can provide insight to help balance aggressive surgical cytoreduction (i.e. supramarginal resection) with sparing functional brain tissue (69).

### Treatment assessment: immunotherapies

The profound inter- and intratumoral heterogeneity in GBM make it challenging to assess treatment response to novel therapeutics. The results of almost all GBM clinical trials have been deemed underwhelming because, on average, patient outcomes are not significantly improved. However, there may be small groups of patients who are responding to these experimental therapies but remain undetected when averaged together with such large, diverse cohorts. Worse yet, treatment response can appear the same or very similar to tumor progression on routine clinical imaging, particularly in the case of immunotherapies. This imaging ambiguity creates a major challenge in clinical decision-making, as it is unclear whether the immunotherapy is working or a change in treatment is warranted. There are numerous documented cases of such circumstances in which surgical biopsies reveal treatment-related immune infiltrate in the absence of tumor cells (73).

**Figure 3:**
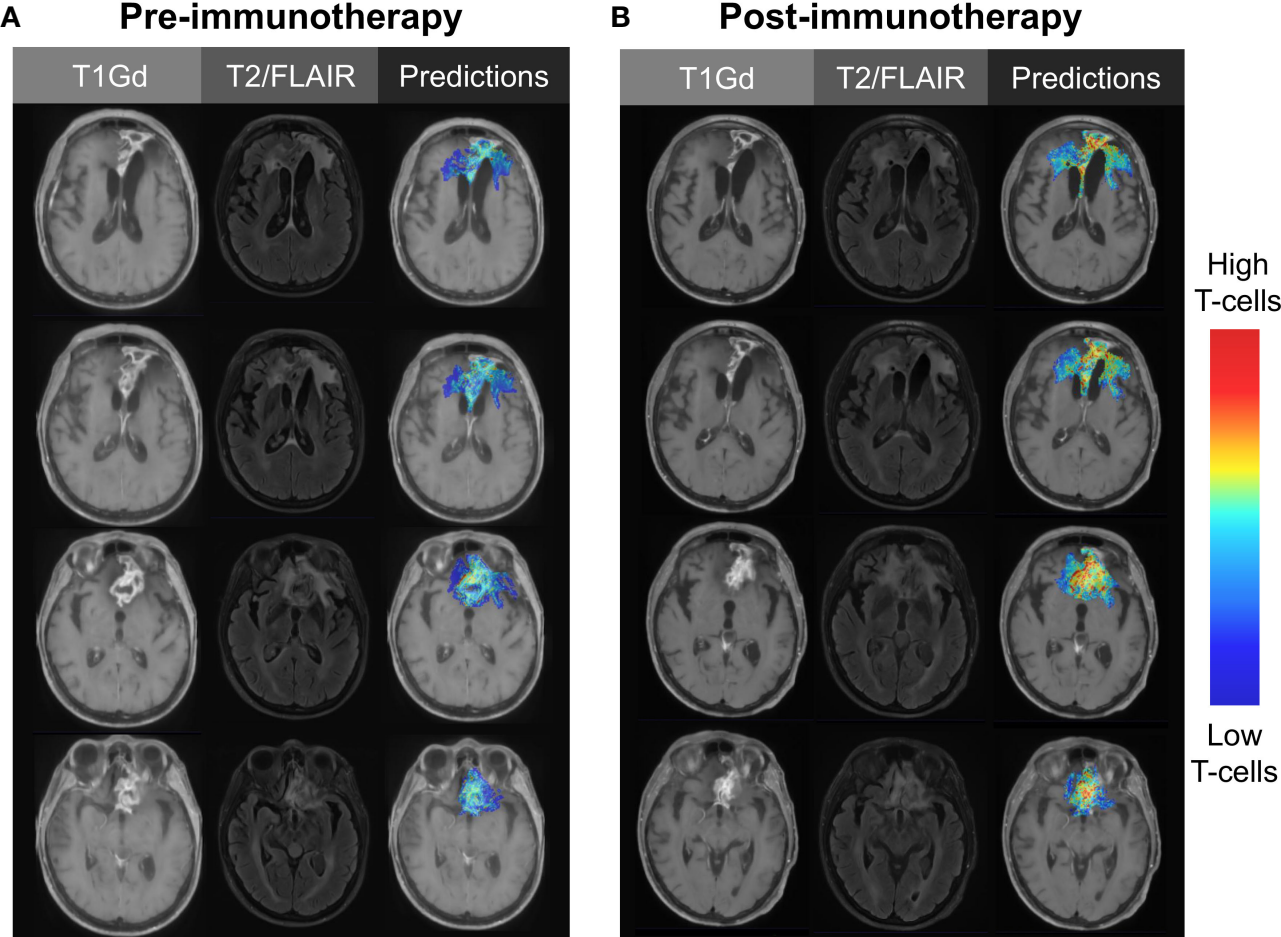
Illustrative case using image-based modeling to predict intratumoral T-cell abundance **(A)** before and **(B)** after the initiation of immunotherapy. Serial predictions over time may aid in the evaluation of novel immunotherapies, as treatment-responsive patients may go undetected with traditional methods of clinical trial assessment.

Image-based machine learning models offer us the opportunity to identify groups of patients who responded to therapies through a targeted, hypothesis-driven approach. For example, many immunotherapies are anticipated to boost a patient’s systemic and intratumoral T-cell response. T-cell abundance can be estimated through a variety of means (e.g., flow cytometry, immunohistochemistry, RNA sequencing followed by deconvolution) and used as the biological target variable in the generation of radiomics models. Image-based models, trained on T-cell estimates from samples paired with image-localized biopsies, can be applied to MRIs from immunotherapy trials to measure the changes in predicted intratumoral T-cell dynamics over the course of therapy (**Figure 3**) (29). The ultimate long-term goal of this approach is to identify patients who responded or are responding to novel therapies. This identification will allow us to effectively streamline targeted therapies and move us closer towards a reality of individualized medicine.

## Conclusions

Image-based models have the potential to transform the way we evaluate CNS tumors, prognosticate patient outcomes, and even assess the efficacy of novel therapeutics. While whole-tumor classification models have gained popularity and certainly hold value, the innate intratumoral heterogeneity of these malignancies requires a spatial, voxelwise approach to truly assess the entire landscape. The collection of image-localized biopsies in the operating room can be resource-intensive, and it will require massive amounts of data collection to validate and apply these spatiotemporal machine learning approaches on a large scale. However, this effort will pay dividends when physicians can non-invasively assess a patient’s dynamic tumoral and microenvironmental landscape in real-time and make personalized treatment decisions accordingly.

## Data availability statement

The original contributions presented in the study are included in the article/supplementary materials, further inquiries can be directed to the corresponding author. There are no primary data or analyses associated with this paper, but inquiries regarding the potential applications we highlighted in the figures can be directed to the corresponding author.

## Author contributions

KS and LH developed the protocols that guided this work. KS provided funding. KB wrote the manuscript and created the figures under the guidance of KS and JR. LC, SR, and AA edited the manuscript and contributed to the figures. All authors contributed to the article and approved the submitted version.
